# Therapeutic efficacy of artemether-lumefantrine in uncomplicated falciparum malaria in India

**DOI:** 10.1186/1475-2875-8-107

**Published:** 2009-05-19

**Authors:** Neena Valecha, Prakriti Srivastava, Suman S Mohanty, Pooja Mittra, Surya K Sharma, Prajesh K Tyagi, Khageswar Pradhan, Vas Dev, Ruchi Singh, Aditya P Dash, Yagya D Sharma

**Affiliations:** 1 National Institute of Malaria Research (NIMR), 22, Sham Nath Marg, Delhi – 110054, India; 2 National Institute of Malaria Research (Field Station), Rourkela, Orissa, India; 3 All India Institute of Medical Sciences, Ansari Nagar, New Delhi, India; 4 National Institute of Malaria Research (Field Station), Guwahati, Assam, India

## Abstract

**Background:**

Artemisinin-based combination therapy (ACT) is the treatment of choice for uncomplicated falciparum malaria. Artemether-lumefantrine (AL), a fixed dose co-formulation, has recently been approved for marketing in India, although it is not included in the National Drug Policy for treatment of malaria. Efficacy of short course regimen (4 × 4 tablets of 20 mg artemether plus 120 mg lumefantrine over 48 h) was demonstrated in India in the year 2000. However, low cure rates in Thailand and better plasma lumefantrine concentration profile with a six-dose regimen over three days, led to the recommendation of higher dose globally. This is the first report on the therapeutic efficacy of the six-dose regimen of AL in Indian uncomplicated falciparum malaria patients. The data generated will help in keeping the alternative ACT ready for use in the National Programme as and when required.

**Methods:**

One hundred and twenty four subjects between two and fifty-five years of age living in two highly endemic areas of the country (Assam and Orissa) were enrolled for single arm, open label prospective study. The standard six-dose regimen of AL was administered over three days and was followed-up with clinical and parasitological evaluations over 28 days. Molecular markers *msp*-*1 *and *msp*-2 were used to differentiate the recrudescence and reinfection among the study subjects. In addition, polymorphism in *pfmdr*1 was also carried out in the samples obtained from patients before and after the treatment.

**Results:**

The PCR corrected cure rates were high at both the sites viz. 100% (n = 53) in Assam and 98.6% (n = 71) in Orissa. The only treatment failure case on D7 was a malnourished child. The drug was well tolerated with no adverse events. Patients had pre-treatment carriage of wild type codons at positions 86 (41.7%, n = 91) and 184 (91.3%, n = 91) of *pfmdr1 *gene.

**Conclusion:**

AL is safe and effective drug for the treatment of acute uncomplicated falciparum malaria in India. The polymorphism in *pfmdr*1 gene is not co-related with clinical outcome. However, treatment failure can also occur due to incomplete absorption of the drug as is suspected in one case of failure at D7 in the study. AL can be a viable alternative of artesunate plus sulphadoxine/pyrimethamine (AS + SP), however, the drug should be used rationally and efficacy needs to be monitored periodically.

## Background

Resistance in *Plasmodium falciparum *to commonly used anti-malarial drugs, especially chloroquine, is being increasingly recognized in India [1]. More than 80% of the therapeutic efficacy studies (n = 143) conducted from 2001–07 indicate failure to chloroquine beyond cut off level of 10% (unpublished data). WHO recommends use of artemisinin-based combination therapy (ACT) to counter the development of resistance in *P. falciparum *to anti-malarials and to achieve rapid resolution of parasitaemia and morbidity [2]. Accordingly, the National drug policy has been revised in India in favor of using ACT. Although at present artesunate plus sulphadoxine/pyrimethamine (AS+SP) has been recommended for the treatment of uncomplicated falciparum malaria, data are also being generated for new fixed dose combinations to help the National Programme to adopt the most effective, easy to administer, feasible, safe and cost-effective strategy.

Artemether-lumefantrine (AL) is a co-formulation of artemether and lumefantrine (an aryl alcohol related to quinine, mefloquine and halofantrine). The reported 28 day cure rate for six- dose regimen ranges between 89.6 and 98.5% and 42 day cure rate ranges between 87 and 94.8% [3-5]. In India, 28 day cure rate of 94.5% was reported by Kshirsagar *et al *[6] with four dose regimen (4 × 4 tablets of 20 mg artemether plus 120 mg lumefantrine over 48 h) in a double-blind comparative study with chloroquine in falciparum malaria. However, in Thailand this regimen resulted in failure rates of 20%, which reduced to 5% by using a six-dose regimen [7]. Therefore, high efficacy observed earlier in South-East Asia (SEA) with low dose, including India, may be due to background immunity which may not be sustainable. In addition, the better plasma lumefantrine concentration profiles have been observed with high dose regimen [8].

The present study was conducted to evaluate the efficacy of AL with six-dose regimen in two endemic regions of India following the WHO (2003) therapeutic efficacy protocols [9].

## Methods

### Study site and design

**Figure 1:**
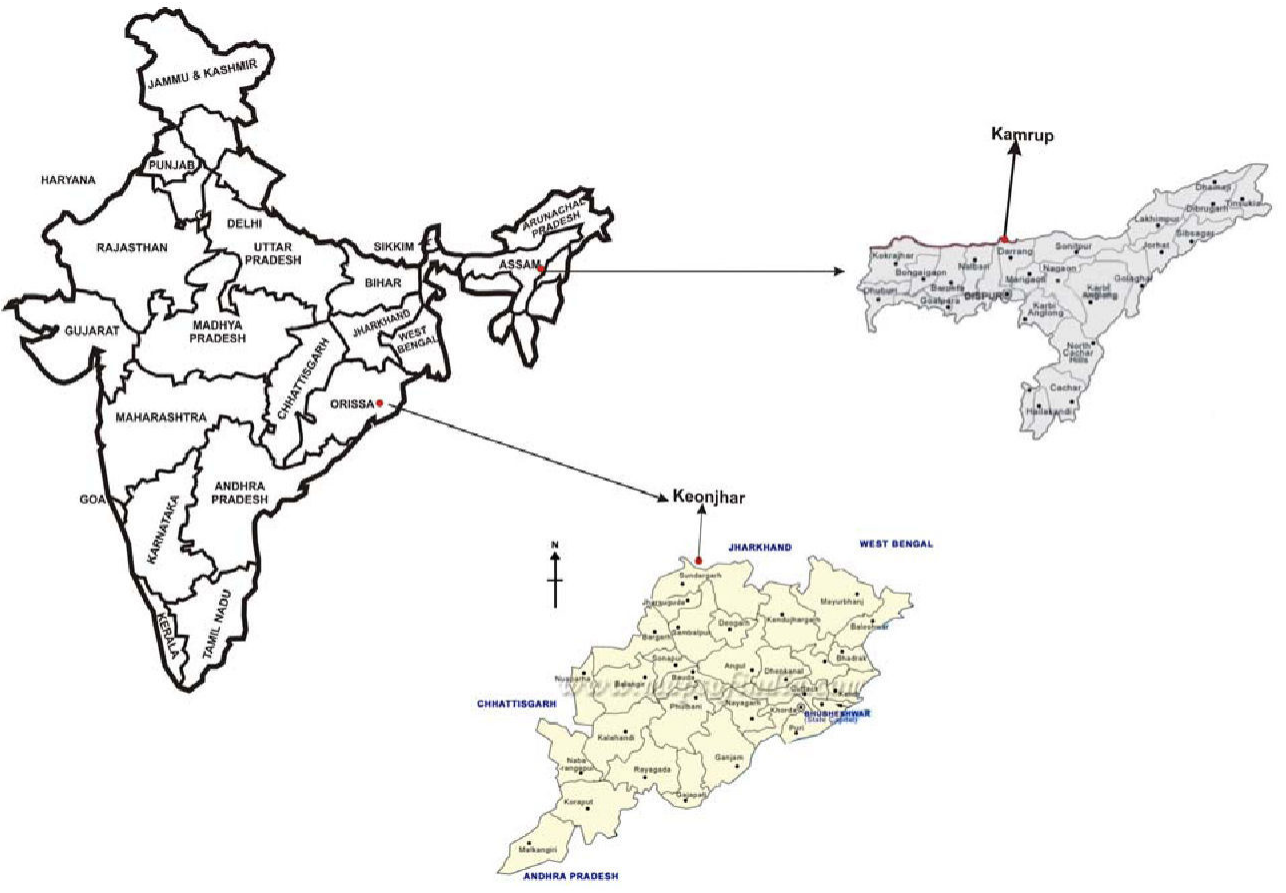
**Study sites**.

The study was conducted in Kamrup district (Assam) in north-eastern India and Keonjhar district (Orissa) in eastern part of India (Figure 1), where the annual parasite incidence in 2006 were 2.7 and 17.5, respectively. At both the sites, malaria transmission is perennial with the predominance of falciparum malaria (76.3% in Kamrup and 96.5% in Keonjhar).

It was an open label, single arm prospective trial based on the therapeutic efficacy protocols of WHO [9]. The primary end point was 28-day cure rate. Secondary end points included proportion of aparasitaemic patient on days 1, 2 and 3 and post-treatment gametocyte carriage. The study was approved by the Ministry of Health, Govt. of India and the Ethics Committee of National Institute of Malaria Research.

### Patients

Patients with microscopically confirmed *P. falciparum *malaria (asexual parasites 1,000–100,000/μl), with fever (axillary temperarture, ≥ 37.5°C) or history of fever in preceeding 24 h, who gave voluntary consent were enrolled in local clinics. Pregnant or lactating women and children under 5 kg bodyweight were excluded. Patients with other febrile conditions, danger signs [9] or severe malaria were also excluded.

### Procedures

After obtaining the informed written consent from patients or guardian, a medical history including presenting symptoms, current medications and previous anti-malarial use was obtained. A complete physical examination was performed and case record form was completed for each patient. Clinical history, examination and other investigations were all recorded. Blood was collected for parasitology and molecular biology studies.

### Treatment

Patients were given AL (Coartem^®^, Novartis Pharmaceuticals Corporation, Suffern, New York, USA; Batch No.: F0443) according to body weight with biscuits and glass of water. Patients weighing 10–15 kg received one tablet (20 mg artemether plus 120 mg lumefantrine) per dose, those weighing 15–25 kg received two, those of 25–35 kg received three, and those >35 kg received four tablets. In total, six doses were administered at hours 0, 8, 24, 36, 48, and 60. Treatment with both the doses, every day, was directly observed by the study team. No patient had vomiting and, therefore, re-administration of the drug was not required.

### Follow-up

All the patients were followed-up on days 1, 2, 3, 7, 14, 21 and 28. On each day, in addition to physical examination, blood smears for malaria parasites and filter paper spot samples for genotyping were obtained.

### Laboratory methods

Parasite counts were done on Giemsa-stained thick films and the number of parasites per 200 WBC was counted by light microscopy, by microscopists with 10–15 years of experience. Assuming a WBC count to be 8,000/μl, parasitaemia was calculated and expressed as per μl. A thick smear was regarded as negative on initial review if no parasites were seen in 100 high power fields and 10% of positive and negative slides were cross-checked.

### Polymerase chain reaction and nucleotide sequencing

Genomic DNA of parasite was isolated from filter paper using QIAamp DNA Blood kit (Qiagen, Valencia, California, USA) according to the manufacturer's instructions and used for a polymerase chain reaction (PCR). To distinguish between recrudescent and new infections PCR method was used to analyse polymorphisms in the merozoite surface protein (MSP) genes, *msp-1 *and *msp-2 *in pre-treatment and post-treatment isolates following the method described earlier [10].

Two fragments of *pfmdr1*, one spanning codons 71–242, and other covering 945–1308 codons were amplified to analyze SNP's at codons 86, 184, 1034, 1042 and 1246. In *pfcrt *SNP's at codons 72–76 were studied by amplifying region-spanning codons 44–177. Primers and cycling conditions used for amplification of fragments of *pfmdr*1 are shown in Table 1, while the strategy followed for *pfcrt *analysis was same as described earlier [11]. The PCR products were purified from the agarose gel by using gel extraction Kit **(**Mdi, Membrane Technology, India). About 50–250 ng of the purified DNA was used for sequencing PCR using ABI Big Dye Terminator Reaction Ready Kit Version 3.1(PE Applied Biosystems, California, USA). The sequencing PCR was performed in a volume of 20 μl with 1 μl of Terminator Ready Reaction Mix (TRR), 3.2 pmol of gene-specific primer. Cycling conditions for the sequencing PCR included 25 cycles of denaturation at 96°C for 10 sec, annealing at 50°C for 5 sec, and extension at 60°C for 4 min. Templates were purified and sequenced on an ABI Prism 310 Genetic Analyzer (PE Applied Biosystems).

**Table 1:** Primer sequences and PCR conditions for *pfmdr1 *amplification

Fragment amplified	Primer name	Product size (bp)	Primer sequence	Cycling conditions*
Pfmdr1 fragment 1 (codons 86&184)	Pf 86	677	AGAGGTTGAAAAAGAGTTGAAC	94°C for 30 sec; 56°C for 45 sec; and 72°C for 1 min, 40 cycles
	Pr 86		TTCTTATTCCCATTAAAGCCTC	
	Nf 86	471	CCGTTTAAATGTTTACCTGCAC	94°C for 30 sec; 55°C for 1 min;
	Nr86		AACGCAAGTAATACATAAAGTCA	and 72°C for 30 sec, 30 cycles
Pfmdr1 fragment 2	Pf 1034	1092	GTGTATTTGCTGTAAGAGCTAG	94°C for 30 sec; 57°C for 1 min;
(codons1034,1042&1246)	Pr 1034		CATATGGTCCAACATTTGTATC	and 72°C for 1 min, 40 cycles
	Nf1034	950	GATGAAATGTTTAAAGATCCAAG	94°C for 30 sec; 56°C for 1 min;
	Nr1034		TCATCTATAGCAGCAAACTTAC	and 72°C for 1 min, 35 cycles

Note: *In each PCR, initial denaturation was at 94°C for 15 min, and final extension at 72°C for 15 min

### Statistical analysis

Data was entered in WHO TM software. The cumulative risk of failure was assessed by survival analysis with the Kaplan Meier method.

## Results

**Figure 2:**
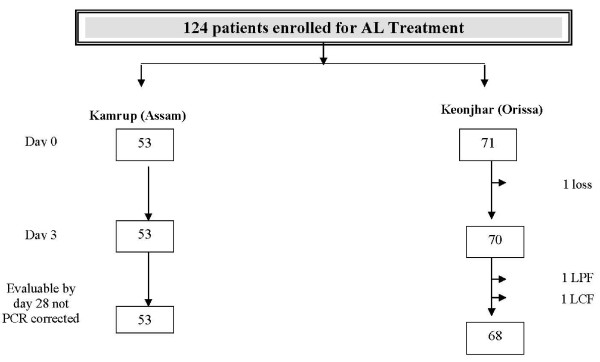
**Flow chart – LPF – Late Parisitological Failure; LCF – Late Clinical Failure**.

**Figure 3:**
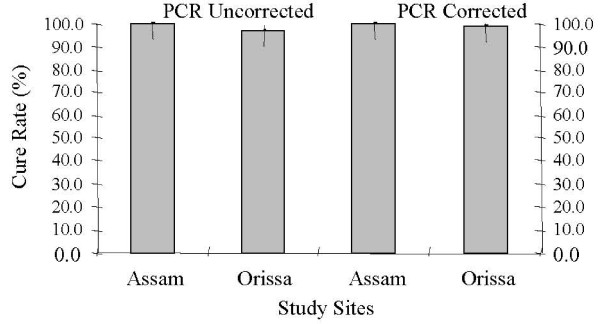
**PCR uncorrected and PCR corrected cure rates by per protocol analysis**.

Patients were screened between July and October 2007 at Kamrup District, Assam and August to October 2007 at Keonjhar District, Orissa. A total of 124 patients were enrolled. Baseline characteristics and age category is shown in table 2. One patient was lost to follow-up due to movement away from site and could not be traced. One was withdrawn on D28 since it was classified as reinfection by PCR. End point was reached in 122 cases (Figure 2). No discrepancies (>30% variation in count) were found on crosschecking. Response to treatment was classified according to WHO guidelines into early treatment failure (ETF), late treatment failure (LTF) and adequate clinical and parasitogical response (ACPR). PCR-corrected and uncorrected cure rates were determined by per protocol method using WHO software. In addition, Kaplan Meier analysis was done where loss to follow-up, withdrawals or reinfections were censored on last day of follow-up. The PCR-uncorrected cure rates by per protocol analysis was 100% and 97.1% in Assam and Orissa respectively (Figure 3). The parasite clearance was rapid at both the sites and complete clearance of parasiaemia within 48 h was observed in 79.2 and 90.1% patients in Assam and Orissa, respectively. The mean parasite clearance time (PCT) was 50.8 ± 11.2 h and 34.1 ± 16.1 h in Assam and Orissa respectively. Proportion of patients with gametocytes at enrollment was 18.8% and 34.2% respectively in Assam and Orissa and it decreased to 1.8% and 11.4% respectively by D7. On D14 only one patient in Orissa had gametocytes and by D28, all patients were without gametocytes except the one who had treatment failure on D7 in Orissa. Two patients who reported with recurrent parasitaemia were treated with AS+SP in standard doses as recommended in the National Drug Policy. Out of these two treatment failure cases in Orissa, genotyping using *msp-1 *and *msp-2 *confirmed one of them as reinfection and one as recrudescence. This led to PCR corrected cure rates of 98.6% in Orissa. The cumulative risk of failure (PCR corrected) by Kaplan Meier analysis was 0.014 (95% CI 0.002–0.097) in Orissa while it was zero in Assam. The response was similar in all age categories and the only patient who had recrudescence on D7 was a seven year-old child, weighing 11 kg. The parasite count on D7 was 48/μl (asexual parasites with gametocyte count of 192/μl) in this patient. PCR analysis confirmed that it was a single clone infection and D0 and D7 samples displayed the matching allele bands indicating a case of recrudescence. Molecular analysis of *pfmdr*1 revealed 86Y in both D0 and D7 samples of this patient. *Pfcrt *haplotype as determined by DNA sequencing was C_72_M_74_N_75_K_76 _in both D0 and D7 samples reiterating the case as recrudescence.

**Table 2:** Demographic and clinical characteristics of patients on inclusion

State (District)	Assam (Kamrup)	Orissa (Keonjhar)
No. of cases enrolled	53	71

Gender ratio (M/F)	36/17	32/39

Age category	Mean ± SD	Mean ± SD
<5 Year	3.3 ± 0.5 (n = 3)	3.2 ± 0.9 (n = 13)
5–15	8.6 ± 2.7 (n = 24)	8.2 ± 2.8 (n = 28)
Adult	29.7 ± 11.2 (n = 26)	25.3 ± 10.2 (n = 30)
**All**	**15.0 ± 14.3 (n = 53)**	**11.1 ± 12.14 (n = 71)**

% patients with fever on D0	66.04	100

Parasitaemia/μl (Geometric Mean)	7922.7	7653.3
Range	1000 – 76190	1040 – 99200

### Genotype analysis of the *pfcrt *and *pfmdr1 *gene

A total of 124 clinical isolates (53 from Assam and 71 from Orissa) were analysed for genetic polymorphism in *pfmdr*1 and *pfcrt *genes. In *pfmdr*1, 58 isolates (26 from Assam and 32 from Orissa) could be analyzed for both the fragments. Fragment 1 covering codons 86 and 184 was amplified from 91 isolates (27 in Assam and 64 in Orissa) while only 63 isolates (36 from Assam and 27 from Orissa) were successfully amplified and sequenced for *pfcrt *region covering codons 72–76. Out of the five *pfmdr1 *codons analysed (N86Y, Y184F, S1034C, N1042D and D1246Y), mutation was observed only at 86 and 184 codons while others remained wild type among all isolates. There were a total of 3 different *pfmdr*1 alleles among the isolates. Genotype **Y**_**86**_Y_184_S_1034_N_1042_D_1246 _was more prevalent (62.06%, total n = 58) than wild type (N_86_Y_184_S_1034_N_1042_D_1246_) and the single mutant (N_86_**F**_**184**_S_1034_N_1042_D_1246_) alleles which were present in 31.03 and 6.89% of isolates, respectively. At codon 86, pre-treatment carriage of N86 was 41.7% while 86Y was 58.3%. However, at codon 184 most of the isolates showed 184Y (91.3%, total n = 91). Mutation in *pfcrt *was observed at 72 (14.89% n = 9), 74(68.6%, n = 43), 75 (68.6% n = 43) and 76 (70% n = 53) codons among 63 isolates with C_72_**I**_**74**_**E**_**75**_**T**_**76 **_triple mutant allele being most predominant (68.6%) while isolates with C_72_M_74_N_75_**T**_**76 **_were rare (1.4%). Only 14.9% isolates were found to contain the wild type C_72_M_74_N_75_K_76_or double mutant **S**_**72**_M_74_N_75_**T**_**76**_alleles.

### Regional prevalence of *pfmdr1 *and *pfcrt *alleles

All the three genotypes of *pfmdr*1 were present in both Assam (n = 26) and Orissa (n = 32). Single mutant **Y**_**86**_Y_184_S_1034_N_1042_D_1246 _genotype was more prevalent in both regions as compared to N_86_Y_184_S_1034_N_1042_D_1246 _and N_86_**F**_**184**_S_1034_N_1042_D_1246 _genotypes (Table 3). *pfcrt *triple mutant (C_72_**I**_**74**_**E**_**75**_**T**_**76**_) genotype was present in highest proportion in both Assam (72.2% n = 36), and Orissa (64.2%, n = 27). Proportionately wild type C_72_M_74_N_75_K_76_genotype was more prevalent in Orissa (25%) than in Assam (8.3%). Also single mutant C_72_M_74_N_75_**T**_**76 **_was found only in Orissa (3.5%). **S**_**72**_M_74_N_75_**T**_**76 **_genotype was present in both Assam (19.4%) and Orissa (7.1%).

**Table 3:** Regional distribution of *pfcrt *and *pfmdr*1 genotypes

		**Isolates, no. (%)**		
Genes	Genotypes	Assam (n = 26)	Orissa (n = 32)	Total (n = 58)

***pfmdr*1**				

Wild-type	N_86_Y_184_S_1032_N_1042_D_1246_	7 (26.9%)	11 (34.3%)	18 (31.03%)

Single-mutant	**Y**_**86**_Y_184_S_1032_N_1042_D_1246_	18 (69.2%)	18 (56.2%)	36 (62.06%)

Single-mutant	N_86_**F**_**184**_S_1032_N_1042_D_1246_	1 (3.8%)	3 (9.3%)	4 (6.89%)

***pfcrt***		**Assam (n = 36)**	**Orissa** **(n = 27)**	**Total** **(n = 63)**

Wild-type	C_72_M_74_N_75_K_76_	3 (8.3%)	7 (25.9%)	10 (15.8%)

Single-mutant	C_72_M_74_N_75_**T**_**76**_	-	1 (3.7%)	1 (1.5%)

Double-mutant	**S**_**72**_M_74_N_75_**T**_**76**_	7 (19.4%)	2 (7.4%)	9 (14.2%)

Triple-mutant	C_72 _**I**_**74**_**E**_**75**_**T**_**76**_	26 (72.2%)	17 (62.9%)	43 (68.2%)

Note: Mutated amino acids are in bold

### Paired sample analysis

In this study, two treatment failure cases were recorded in Orissa and *msp*-1 and *msp*-2 genotyping confirmed one of them as reinfection and other as a recrudescence. Another case in Orissa, reported on D38 with positive parasitaemia which was reinfection and as such not included in analysis of 28 day failure rate. Out of these cases of reinfection, one had **Y**_**86**_Y_184_S_1034_N_1042_D_1246 _and another showed N_86_Y_184_S_1034_N_1042_D_1246 _haplotype at D0. However, on the day of failure, both cases had different *pfmdr1 *genotypes, N_86_Y_184_S_1034_N_1042_D_1246 _(D28) and N_86_**F**_**184**_S_1034_N_1042_D_1246 _(D38) respectively (Table 3). Genotype analysis of *pfcrt *at D0, for both isolates was C_72_**I**_**74**_**E**_**75**_**T**_**76 **_but in failure case at D28 it was C_72_M_74_N_75_**T**_**76 **_and at D38 it was mixture of C_72_M_74_N_75_**T**_**76 **_and C_72_**I**_**74**_**E**_**75**_**T**_**76 **_genotypes. In the recrudescence case, the genotype for both *pfmdr1 *and *pfcrt *at D0 and at D7 remained the same i.e.**Y**_**86**_Y_184_S_1034_N_1042_D_1246 _and C_72_M_74_N_75_K_76 _respectively (Table 4).

**Table 4:** Genotype analysis of *pfmdr*1 and *pfcrt *in reinfection vs. recrudescences after AL treatment

		**Genotype at Day 0**		**Genotype at Day of failure**	
		
Isolate	Day failure	*pfmdr1*	*pfcrt*	*pfmdr1*	*pfcrt*
Reinfection (n = 2)					

1	Day28	**Y**_**86**_Y_184_S_1032_N_1042_D_1246_	C_72_**I**_**74**_**E**_**75**_**T**_**76**_	N_86_Y_184_S_1032_N_1042_D_1246_	C_72_M_74_N_75_**T**_**76**_

2	Day38	N_86_Y_184_S_1032_N_1042_D_1246_	C_72 _**I**_**74**_**E**_**75**_**T**_**76**_	N_86_**F**_**184**_S_1032_N_1042_D_1246_	C_72_M_74_N_75_**T**_**76**_/C_72 _**I**_**74**_**E**_**75**_**T**_**76**_

Recrudescence (n = 1)					

1	Day7	**Y**_**86**_Y_184_S_1032_N_1042_D_1246_	C_72_M_74_N_75_K_76_	**Y**_**86**_Y_184_S_1032_N_1042_D_1246_	C_72_M_74_N_75_K_76_

Note: Mutated amino acids are in bold

## Discussion

In India, ACT has been introduced in all Primary health centers (PHCs) of 117 high risk districts and 256 chloroquine resistant PHCs of 48 districts for the treatment of falciparum malaria. The ACT recommended by the National Programme is AS+SP, which is highly effective but available as blister pack and not a fixed dose formulation. AL is the only fixed dose combination that has been approved for marketing recently and there is no data on safety and efficacy of recommended six-dose regimen in India. The present study was carried out to assess the efficacy of AL in India and its correlation with molecular markers. The drug was well tolerated and produced rapid parasite clearance. Majority of patients, *viz*. 79.2 and 90.1% in Assam and Orissa respectively, were free of parasites within 48 h and cure rates were high at these sites where chloroquine resistance is high.

A case of treatment failure on D7 has been observed for the first time with the six-dose regimen of AL in the study. There is only one earlier report of failure on D9 in a three-year old child in Comoros [12]. Treatment failure to any drug can be due to true resistance of parasite to drug or inadequate blood levels due to low absorption or altered pharmacokinetics of the drug. Molecular genotyping confirmed recrudescence, which was reiterated by *pfcrt *haplotype C_72_M_74_N_75_K_76 _and *pfmdr*1 **Y**_**86**_Y_184_S_1032_N_1042_D_1246 _haplotype in both D0 and D7 Samples. *In vivo *exposure to AL props up the selection of *pfmdr1 *86N in new inoculation and is suggested to be associated with tolerance to lumefantrine [4]. However, in this case presence of 86Y ruled out the true drug resistance as the cause of treatment failure. Complete intake of therapeutic dose was ensured by supervised therapy, though blood drug concentration was not determined. The dose was also adequate according to body weight of the child, which was low for his age indicating malnutrition. Artemether component is absorbed rapidly, while absorption of lumefantrine is dependent on co-administration with fat. As fatty food is generally not well tolerated by febrile children, the drug was administered with biscuits and water. Malabsorption syndrome, which could lead to incomplete absorption of drug in the patient, was excluded by negative history of chronic diarrhoea or vomiting. Altered distribution or increased clearance may have led to sub-therapeutic levels of drug leading to treatment failure. As such malnutrition has been shown to cause larger volume of distribution causing lower plasma concentration and hypoalbuminaemia may also lead to increased levels of unbound drug resulting in increased metabolic clearance [2].

The likelihood of resistance to AL in India is low, since the drug is not included in the National Drug Policy for the treatment of malaria till date. However, recently, marketing permission has been given to few companies. In this scenario, the reappearance of parasites on D7 led us to investigate the possible misuse of the drug. Six brands of AL were available in the town since 2006 and the drug was dispensed without prescription by the local chemists. Discussion with doctors also revealed that drug was being prescribed in various private clinics. This case highlights the need to study the kinetics of drug in malnutrition. In addition, over the counter availability and possible misuse of the drug raises concern of accelerating resistance in near future to one of the valuable ACT.

In addition to clinical outcomes, pre-treatment prevalence of *pfmdr1 *and *pfcr*t alleles in Indian *P. falciparum *isolates was also studied. High prevalence of 184Y (93.3%) in pre-treatment samples was observed. Genotypes indicate that majority of isolates carried **Y**_**86**_Y_184_S_1034_N_1042_D_1246 _(62.06%) followed by N_86_Y_184_S_1034_N_1042_D_1246 _(31.03%) and only 6.8% isolates had N_86_**F**_**184**_S_1034_N_1042_D_1246_genotype. In *pfmdr1 *mutation at only codons 86 and 184 have been found which is similar to earlier report [13].

Previous studies with AL in Africa [14] showed higher baseline predominance of **Y**_**86**_Y_184_Y_1246 _genotype of *pfmdr1*, and selection of N_86_**F**_**184**_D_1246 _genotype after AL treatment. Results of the present study indicate baseline prevalence of **Y**_**86**_Y_184_D_1246 _haplotype in India. Also 6.8% pre-treatment isolates that carry N_86_**F**_**184**_D_1246_genotype were drug sensitive. Two clinical failure cases were classified as reinfection which selected N_86_Y_184_S_1034_N_1042_D_1246_and **Y**_**86**_Y_184_S_1034_N_1042_D_1246 _haplotype, whereas one case of recrudescence has not shown any change in *pfmdr1 *haplotype, i.e*. *it remained as **Y**_**86**_Y_184_S_1034_N_1042_D_1246_. Since there were very few clinical failure cases, it is difficult to conclude any specific selection of a particular genotype after AL treatment. Presence of *pfcrt *C_72_**I**_**74**_**E**_**75**_**T**_**76 **_genotype in highest proportion indicates the preexistence of high level of CQ resistance among the parasite population [11].

The main limitation of the study is that co-relation of molecular markers with treatment failure was difficult due to very low number of treatment failure cases and drug concentration in this case could not be done.

Considering the prevalence of molecular markers of resistance to sulphadoxine/pyrimethamine (SP) especially in north-eastern states in India [15], the non-availability of fixed dose combination of AS+SP and the high efficacy of AL, the latter can be considered as a good alternative option as ACT for the treatment of falciparum malaria in India. However, adequate care should be taken to ensure compliance and good absorption of the drug.

Lack of correlation of molecular markers for resistance with clinical outcome makes it difficult to monitor and predict resistance to this combination. Efficacy of AL needs to be monitored using *in vivo *studies and further molecular studies may help in developing markers to predict impending resistance.

## Conclusion

Artemether-lumefantrine is safe and effective drug for the treatment of uncomplicated falciparum malaria in India. The efficacy of this ACT needs to be carefully monitored periodically since the treatment failures can occur due to resistance as well as subtherapeutic levels due to inadequate absorption especially if not administered with fat or in malnutrition. As such malnutrition and poor uptake of fat is common in poor tribal population. However, since AL is the only fixed combination available in India till date, it can be a viable alternative to AS+SP for the treatment of uncomplicated falciparum malaria.

## Abbreviations

AL: artemether-lumefantrine; SP: sulphadoxine/pyrimethamine; ACT: artemisinin-based combination therapy.

## Competing interests

The authors declare that they have no competing interests.

## Authors' contributions

NV: PI, Development of protocol, Execution, quality control of study and preparation of manuscript. PS, PM: Molecular studies and Preparation of manuscript. Ruchi Singh: Molecular studies. SSM, PKT and KP: Patient enrollment and Parasitology. SKS and VD: Supervision and Co-ordination of field work. APD: Critical review of manuscript. YDS: Protocol and review for molecular studies and manuscript.
